# Can Mixed Strains of *Lactobacillus* and *Bifidobacterium* Reduce Eczema in Infants under Three Years of Age? A Meta-Analysis

**DOI:** 10.3390/nu13051461

**Published:** 2021-04-25

**Authors:** Minghui Sun, Jing Luo, Hanmei Liu, Yue Xi, Qian Lin

**Affiliations:** Department of Nutrition Science and Food Hygiene, Xiangya School of Public Health, Central South University, 110 Xiangya Rd., Changsha 410078, China; sun.1234@csu.edu.cn (M.S.); luojing2546@csu.edu.cn (J.L.); hanmeiliu@csu.edu.cn (H.L.); summerxi@csu.edu.cn (Y.X.)

**Keywords:** eczema, probiotics, *Bifidobacterium*, *Lactobacillus*, infants, meta-analysis

## Abstract

(1) Background: Whether early supplementation of probiotics to improve intestinal flora can effectively prevent eczema remains a controversial issue. We aimed to investigate the effect of a mixed strain of *Lactobacillus* and *Bifidobacterium* on eczema in infants under three years old at present; (2) Methods: We searched the databases of PubMed, Web of Science, and Cochrane Library, as well as National Knowledge Infrastructure (CNKI), WeiPu (VIP), and WanFang Data (WanFang) for randomized controlled trials (RCTs) of probiotics in the prevention of eczema in infants without language restriction. The main outcome was eczema incidence, while adverse events during the intervention constituted the secondary outcome. The random-/fixed-effects model was utilized to calculate the combined relative risk (RR) and 95% confidence interval (CI). The methodological quality of the study was evaluated using the Cochrane “bias risk” tool. According to the initial intervention time, subgroup analysis was carried out, follow-up time, family history, etc.; (3) Results: Nine articles were selected (2093 infants). The *Lactobacillus* and *Bifidobacterium* mixed strain could prevent eczema in infants under three years of age compared to the placebo (RR = 0.60; I^2^ = 67%; *p* < 0.001). Subgroup analysis revealed that the mixture of two probiotic strains had preventive effects on both infants with positive (RR = 0.53; I^2^ = 52%; *p* < 0.001) and negative (RR = 0.69; I^2^ = 62%; *p* = 0.02) family history; The follow-up time for ≤12 months (RR = 0.65; I^2^ = 12%; *p* = 0.01) and 12–24 months (RR = 0.60; I^2^ = 79%; *p* = 0.003), daily dose of probiotics ≤ 1 × 10^9^ and > 1 × 10^9^ colony forming units all can be effective (*p* < 0.01); Compared with the intervention of infants alone (RR = 0.63; I^2^ = 63%; *p* = 0.29), the effect of probiotics mixture at the beginning of pregnancy was more significant (RR = 0.59; I^2^ = 71%; *p* < 0.001); Except for the mixture of *Lactobacillus rhamnosus* *GG* (*LGG*) and *Bifidobacterium longum* (*B. longum*) (*p* = 0.18), other subgroups of intervention group can play a preventive effect (*p* < 0.05); (4) Conclusions: The mixed strain of *Lactobacillus* and *Bifidobacterium* can effectively reduce the incidence of eczema in infants under three years old. However, further research is needed to fully understand the exact mechanism of their effect on infant eczema.

## 1. Introduction

Eczema is the most common and the earliest chronic allergic skin disease in infants [1]. In recent years, the incidence of eczema in infants has risen twofold to threefold. The incidence of eczema in the world is 15–30%, and 60% of children develop eczema within the first year after their birth [2]. In developed countries, such as those Europe and America, the incidence of eczema is about 2–35.8% [3,4,5]. In New Zealand, up to 40% of infants experience this before reaching 15 months [6]. Infant eczema is more symmetrical and usually appears on the head, limbs, and vulva. Erythema, papules, blisters, erosions, and other types of skin rash appear during the illness. Children feel intense itching, which usually leads to scratching and friction, and then aggravates the disease seriously affecting their quality of life [7]. Studies have shown about 50% of children with eczema continue to suffer from the disease until adulthood and may even develop asthma and allergic rhinitis, which brings a heavy burden to individuals and their families and increases the risk of other allergic diseases [8,9,10]. The etiology of eczema is complicated, its pathogenesis is unclear, and there is no effective treatment. Relevant studies have reported that the occurrence of infant eczema is linked to the variation of the intestinal microbiome in the early stage [11], probiotics, breastmilk feeding [12], the birth season ( spring and summer) [13] can reduce the development of eczema.

Probiotics are defined as “living microorganisms” that are beneficial to the human body. They have the advantages of regulating intestinal flora, improving gastrointestinal function, promoting nutrient absorption, enhancing immunity, and inhibiting bacterial growth [14]. The decreases in the proportion of *Bifidobacteria* in the feces of infants with eczema and the increases in the number of *Faecalibacterium prausnitzii* may cause an abnormal T-helper 2 cell (Th2) response [15,16]. Th2 response is particularly prominent in acute eczema [17]. Probiotics can stimulate regulatory T cells and inhibit Th2 response [18], altering the intestinal flora of patients with eczema and improving eczema symptoms. Many studies have evaluated the potential benefits of probiotics for infant eczema. Cabana [19] and others found that early supplementation of probiotics can reduce infants’ risk of allergic diseases, such as eczema and asthma, in infants. A randomized controlled trial (RCT) in China also showed that early use of probiotics could reduce infant eczema and prevent the atopic processes in allergic disease [20]. In 2015, the World Allergy Organization recommended using probiotics in high-risk pregnant women, high-risk breastfeeding women, and high-risk infants [21]. However, the European Society for Allergy and Clinical Immunology believes that there is insufficient evidence to prove that probiotics can prevent food allergy [22]. The latest meta-analysis also shows that probiotic supplementation for pregnant or lactating women and/or infants has little effect on preventing food allergy in infants, but the evidence is uncertain [23]. Variation in research results is mainly due to the different research designs and conditions, including the composition and duration of use of probiotics [24].

*Lactobacillus* and *Bifidobacterium* are the essential probiotics, widely used in medicine and health, the food industry, animal husbandry, and other fields [25]. Previous studies have shown a significant effect on preventing eczema in infants only when mixed strains (*Lactobacillus* and *Bifidobacterium*) were used [26]. However, there are contradictions between the results of this study and other findings [19,27]. Besides, only a few meta-analyses have explored the preventive effect of mixed strains of *Lactobacillus* and *Bifidobacterium* on eczema in infants under three years old. Therefore, the purpose of this study is to examine further the impact of mixed *Lactobacillus* and *Bifidobacterium* supplementation during pregnancy and early infancy on eczema in infants under three years old in order to increase the existing evidence and provide a basis for clinical practice.

## 2. Materials and Methods

### 2.1. Data Sources and Literature Search

We searched three English databases (PubMed, Web of Science, and Cochrane Library) and three Chinese databases (CNKI: www.cnki.net accessed on 24 April 2021, VIP: qikan.cqvip.com accessed on 24 April 2021, and WanFang: www.wanfangdata.com.cn accessed on 24 April 2021) to find RCTs of probiotics to prevent infantile eczema published before January 2020. The search terms included: “probiotics”, “*Bifidobacterium*”, “*Lactobacillus*”, “eczema”, “dermatitis”, “allergic diseases”, “atopic dermatitis”, “infants”, “children”, and “pregnancy”. We also manually searched for published reviews and their references to identify other studies that may meet the criteria. Our study was registered in PROSPERO (CRD42020159738) on 28 April 2020. The contents of the review followed the preferred reporting items for systematic reviews and meta-analyses [28].

### 2.2. Inclusion and Exclusion Criteria

A study was included if the following criteria were met: (1) RCT; (2) the literature was published before January 2020; (3) the subjects included healthy or pregnant women and infants under three years old with a family history of atopic disease; (4) the intervention group received a mixture of *Lactobacillus* and *Bifidobacterium*, while the control group undertook a placebo intervention, and no other probiotic interventions or no intervention; and (5) the primary outcome was the incidence of infant eczema (diagnostic criteria: the UK working group, Hanifin and Rajka (Hanifin 1980) or based on the diagnosis of physicians [29]).

The exclusion criteria were as follows: (1) the subjects had other types of disease, (2) the probiotics were not mixed strains of *Lactobacillus* and *Bifidobacterium*, (3) the probiotics were taken before the study, (4) duplicate studies.

### 2.3. Quality Assessment

Two authors used the Cochrane collaborative tool to conduct an independent assessment of the bias risk for all studies. The evaluation items included random allocation method, allocation scheme concealment, whether the participants and the investigator adopted the blind method, whether the blind method was used in the result evaluation, data integrity of results, selective report of research results, etc. According to the evaluation results of each independent study, the existing risks were evaluated. The results were designated as “low risk”, “high risk”, or “unknown risk”. Any disagreements in the evaluation process were resolved by the third author.

### 2.4. Data Extraction

The two authors independently extracted the detailed data for each included study based on a pre-designed datasheet. The extracted contents included author, publication year, sample size, family history, intervention and control details, follow-up time, outcome evaluation method, adverse events, and results. The third author ensured the consistency of the data extraction. The primary outcome of the study was the incidence of infant eczema at the end of follow-up, while the secondary outcome constituted adverse events during the intervention period.

### 2.5. Data Synthesis and Statistical Analyses

We used Review Manager (Revman) 5.3 software for data analysis and took RR and 95% CI as the observation indexes. *p*-value < 0.05 was considered to be statistically significant. The random-effects models were used for all analyses and I^2^ statistics for heterogeneity [30]. Moreover, we conducted further subgroup analyses based on the time of follow-up, starting point of intervention, and family history.

If the heterogeneity was very high, we conducted a sensitivity analysis to observe whether the combined results and heterogeneity changed to determine the stability of the results. If more than 10 studies were included, we explored possible publication bias through funnel plots and Egger’s linear regression.

## 3. Results

### 3.1. Search Results

**Figure 1:**
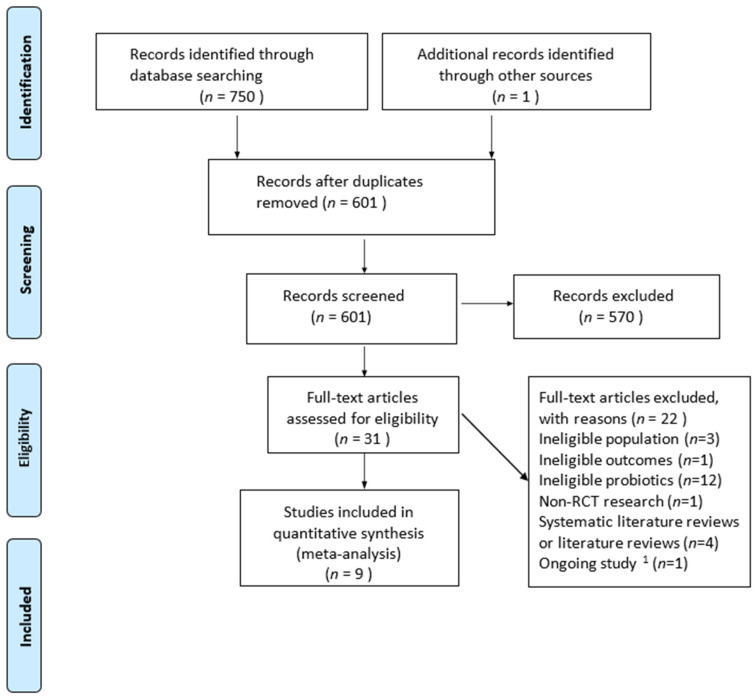
Preferred Reporting Items for Systematic Reviews and Meta-Analyses (PRISMA) flow diagram. ^1^ Ongoing study: the research is still in progress.

We found 750 records through our search strategy (Table S1), and one additional record was identified through a reference. After excluding the duplicate literature, 601 records remained. Another 570 items were removed by reading the title and the abstract. A strict qualification review was conducted on the complete text, and 22 studies were excluded because they did not meet the inclusion criteria. One of the studies is in progress. Finally, this study included nine RCTs [31,32,33,34,35,36,37,38,39] (Figure 1).

### 3.2. Study Characteristics

Table 1 listed the characteristics of the nine qualified RCTs. These studies were published before January 2020. A total of 2093 participants (1051 in the probiotics group and 1042 in the control group) were included in the studies. In all of the studies, the duration of intervention with a mixture of *Lactobacillus* and *Bifidobacterium* varied from pregnancy to the children’s first year. The average follow-up time was 1.5 years. The infants were regarded as “atopic” if they have one or more family members with eczema, asthma, gastrointestinal allergies, allergic urticaria, or allergic rhinoconjunctivitis. Findings from the meta-analyses were evaluated using self-designed questionnaires, follow-up of nurses and doctors, structured interviews related to the symptoms of allergic diseases, physical examination, skin prick test, and blood sampling test. Since Rautava et al. [35] included two groups of patients and supplemented them with different mixtures of *Lactobacillus* and *Bifidobacterium* strains (*LGG* + *B. longum* and *Lactobacillus paracasei* (*L. paracasei*) + *B. longum*), we presented the report twice here.

### 3.3. Bias Risk Assessment

**Figure 2:**
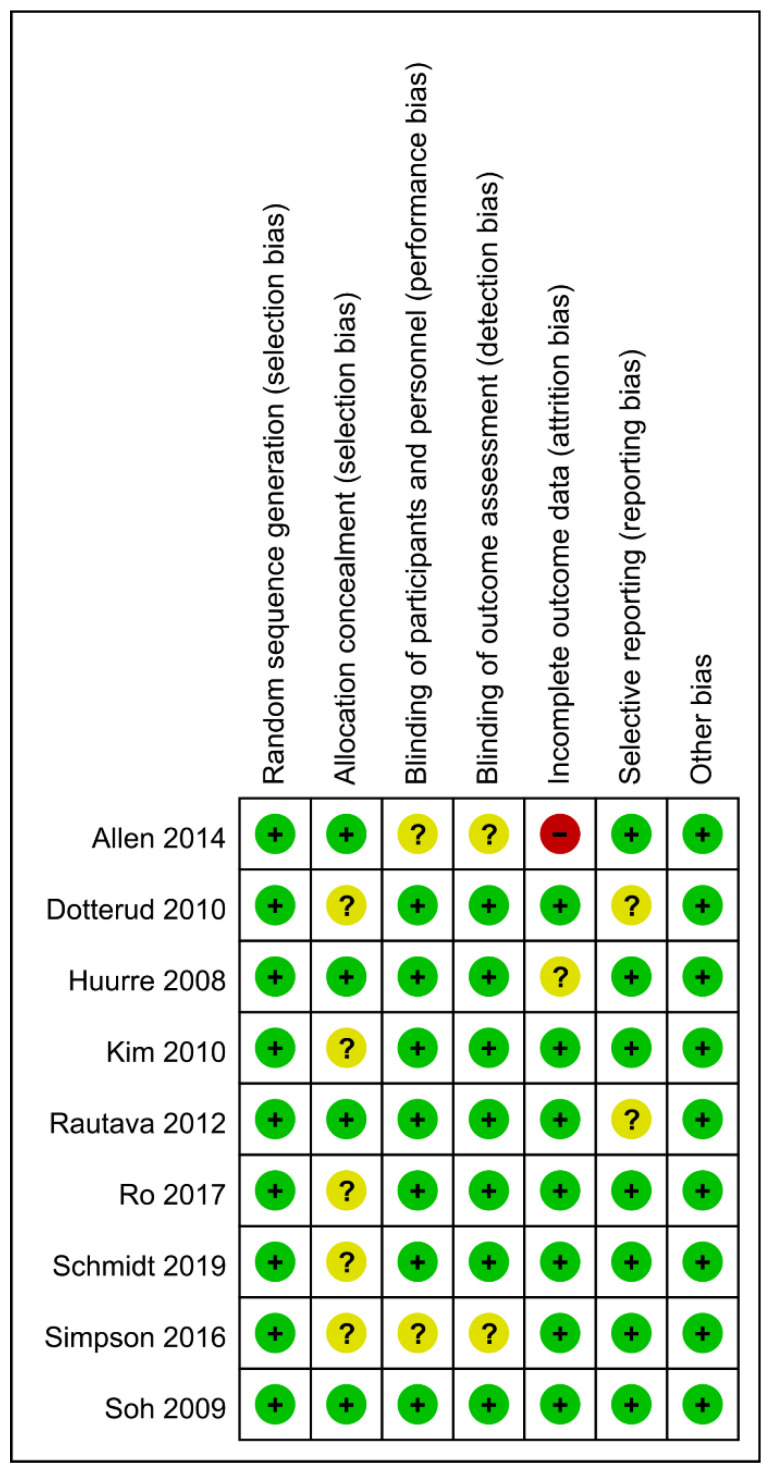
Risk of bias graph per type of bias assessed. “+”: low risk of bias, “-”: high risk of bias, “?”: unknown risk bias.

**Figure 3:**
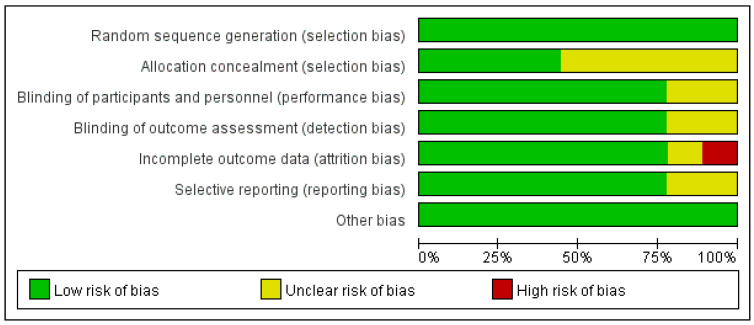
Risk of bias summary for the studies assessed.

The results of Cochrane’s risk of bias assessment (Figure 2 and Figure 3) showed that the overall risk of bias was low. Among them, nine studies described the generation of random sequences in detail, four studies reported proper allocation concealment, seven studies showed that the nature of the product tested (drug or placebo) was unknown to the subjects and the researchers. One study had incomplete results due to missing data. In two studies, it was unclear whether there was reporting bias. Due to the limited number of studies, it was impossible to explore the existence of publication bias.

### 3.4. Effect of Lactobacillus and Bifidobacterium on Prevention of Infant Eczema

**Figure 4:**
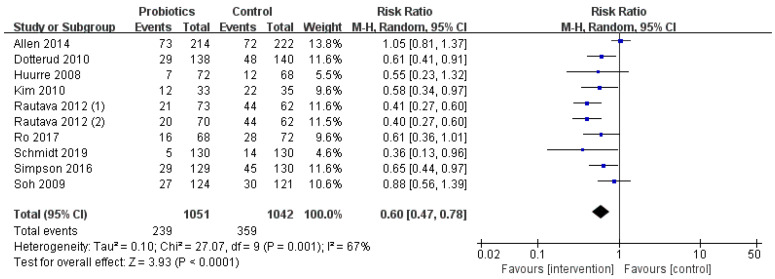
Forest plot of the relationship between *Lactobacillus* and *Bifidobacterium* and infant eczema.

In addition to the trial of Schmidt and Soh et al., other studies were conducted in which mothers began to supplement the mixture of *Lactobacillus* and *Bifidobacterium* during pregnancy. There was high heterogeneity among the studies (I^2^ = 67%, *p* < 0.001), so the random-effects model was used. Fewer infants developed eczema in the probiotics group compared to those in the control group (239 vs. 359), and the RR value of the probiotics group in preventing infant eczema was lower (RR = 0.60; I^2^ = 67%; *p* < 0.001). Due to the high heterogeneity, we conducted a sensitivity analysis and excluded the studies one by one. After further excluding Allen et al.’s [27] and Soh et al.’s [35] study, the combined effect value was still significant (*p* < 0.001) and the heterogeneity decreased from 67% to 0%. Therefore, we determined that these two studies were the source of heterogeneity (Figure 4).

### 3.5. Occurrence of Adverse Events

**Figure 5:**
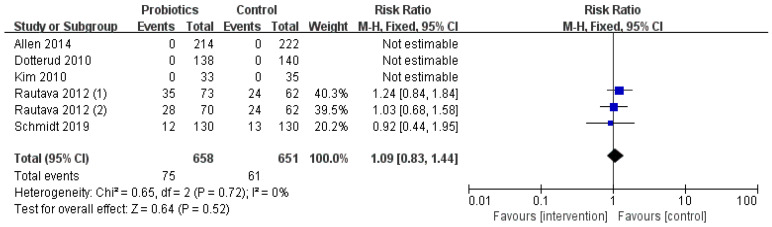
The occurrence of adverse events during the intervention.

The secondary outcome of this study was to compare the adverse events during the intervention period (Figure 5). Overall, five of the included studies reported adverse events and three of them had no adverse events. In the probiotic group, 75 infants had gastrointestinal symptoms and food reactions, while in the control group, 61 cases were reported. There was no significant difference (RR = 1.09; I^2^ = 0%; *p* = 0.52) between the two groups.

### 3.6. Subgroup Analysis Results

#### 3.6.1. Influence of Family History on Occurrence of Infant Eczema

**Figure 6:**
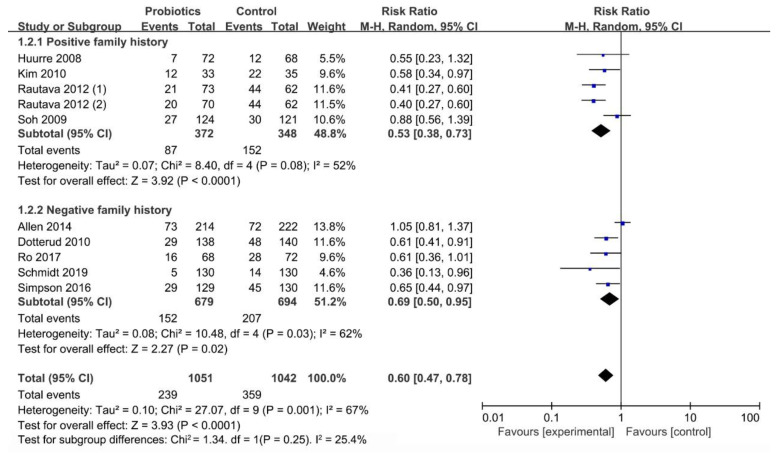
The relationship between mixed strains of *Lactobacillus* and *Bifidobacterium* and infant eczema with different family histories.

The family history-specific sub-meta-analyses showed that the mixed strains of *Lactobacillus* and *Bifidobacterium* have a preventive effect on infants with positive (RR = 0.53; I^2^ = 52%; *p* < 0.001) and negative (RR = 0.69; I^2^ = 62%; *p* = 0.02) family history (Figure 6).

#### 3.6.2. Influence of Initial Time of Intervention on Occurrence of Infant Eczema

**Figure 7:**
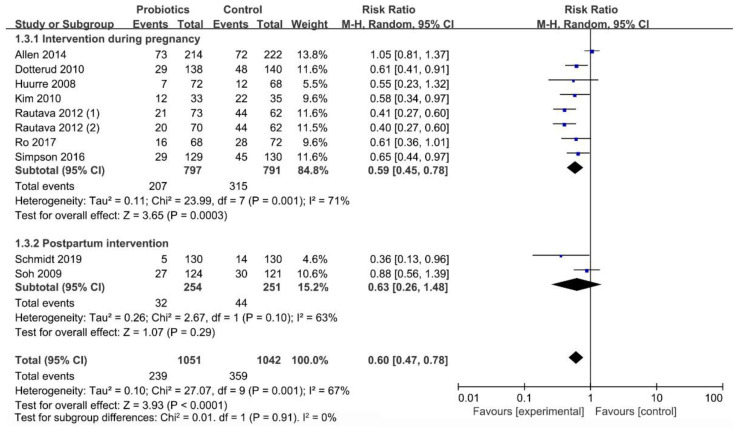
Intervention time subgroup analysis forest plot.

Figure 7 presented an evaluation of the start time among the different interventions. The mothers started taking the supplement composed of the mixed strains of *Lactobacillus* and *Bifidobacterium* during their pregnancy, which had a significant effect on reducing the occurrence of eczema in infants (RR = 0.59; I^2^ = 71%; *p* < 0.001). Conversely, intervention in the infants after delivery did not have a significant effect (RR = 0.63; I^2^ = 63%; *p* = 0.29).

#### 3.6.3. Effect of Follow-up Time (≤12 Months/12–24 Months) on Occurrence of Infant Eczema

**Figure 8:**
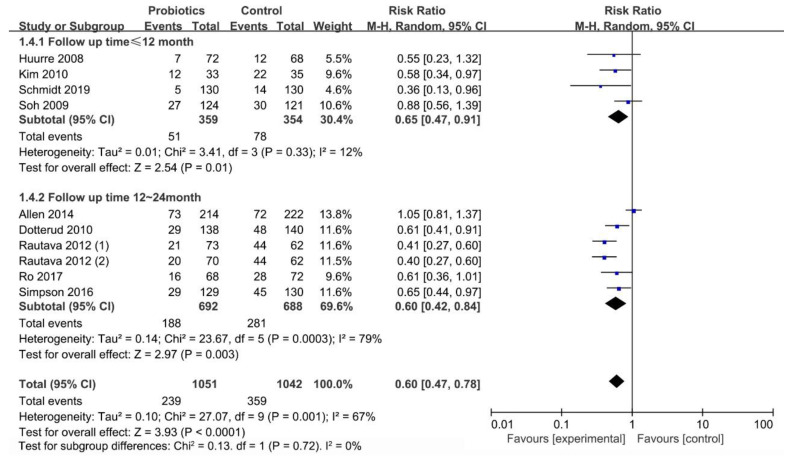
Forest plot of the association between mixed strains of *Lactobacillus* and *Bifidobacterium* and eczema at ≤12 months and 12–24 months of age.

The subgroup analysis of studies with different follow-up times (Figure 8) revealed a significant effect of the mixed strains of *Lactobacillus* and *Bifidobacterium* at ≤12 months (RR = 0.65; I^2^ = 12%; *p* = 0.01) and 12–24 months (RR = 0.60; I^2^ = 79%; *p* = 0.003) after birth.

#### 3.6.4. Effect of Probiotics Dosage and Strains on Occurrence of Infant Eczema

Results of probiotics dosage and strains analysis were indicated in Figures S1 and S2. The range of daily dose of probiotics used in included studies was extensive, ranging from 1 × 10^7^–5 × 10^10^ colony-forming units, but all of them were effective in preventing infant eczema (*p* < 0.01). Except for the mixture of *LGG* and *B. longum* (*p* = 0.18), other subgroups of the intervention group can play a preventive effect (*p* < 0.05). Affected by apparent heterogeneity and fewer documents, more studies are still needed to confirm its effectiveness.

## 4. Discussion

The meta-analysis included a randomized, double-blind, placebo-controlled trial of oral *Lactobacillus* and *Bifidobacterium* mixed strains for pregnant women and/or breastfeeding mothers or infants under three years of age to prevent infantile eczema. The results showed that using the above probiotic mixtures as a supplement can avoid infant eczema (RR = 0.60; 95% CI: 0.47–0.78; *p* < 0.001), which is consistent with the findings of other research [40,41]. Previous studies have confirmed that the intestinal flora of normal children was dominated by *Lactobacillus* and *Bifidobacterium*, while the level of *Clostridium* in children with allergies was significantly increased, and the contents of *Enterococcus* and *Lactobacillus* were reduced markedly. The number of *Escherichia coli* and *Staphylococcus aureus* in the intestines of children with eczema increased, while the number of *Bifidobacteria* and *Lactobacilli* decreased [42]. This may be the reason why *Lactobacillus* and *Bifidobacterium* mixed strains can prevent allergic eczema in children. *Lactobacillus* and *Bifidobacterium* are also recommended internationally to treat children with allergic eczema [43].

The subgroup analysis of different follow-up times revealed that the effect of the mixed strains of *Lactobacillus* and *Bifidobacterium* in preventing infantile eczema was significant, not only within 12 months after birth but also within 12–24 months. Infant allergic diseases mostly occur within three years of age, and the incidence rate is highest at one year old. Most allergic diseases can heal on their own as the infants grow older and their immune systems gradually improve [44]. The mixed use of these two strains could prevent eczema in high-risk infants and the general population, similar to the meta-analysis conducted by Wang Yu et al. [45]. However, Loo et al. found that probiotic supplementation during the first six months of life did not prevent eczema and allergies in high-risk Asian babies [46]. The results obtained were different, probably because of the various types of probiotic strain and the time of follow-up applied in the different studies. There were also different conclusions about the influence of intervention time on the effect of probiotics. In this study, the mothers who started using probiotics as a supplement during their pregnancy to prevent eczema attained a significant effect. However, simply intervening with the baby after delivery did not show any effect. We speculate that this result may be due to pregnancy being a critical period for determining and forming intestinal microbes in infants. Taking probiotic mixtures during pregnancy had a stronger effect on regulating host immunity in the early stage of life. It should be emphasized that our study only included two trials of probiotic supplementation to infants after birth, resulting in a significant difference in the overall number of cases. Thus, the quality of evidence was low and was not sufficient to conclude. Yet, Boyle et al. [39] concluded that prenatal intervention alone has no preventive effect on eczema, asthma, and other food allergies, suggesting the importance of postpartum intervention to prevent allergic diseases.

In addition, we found that *Lactobacillus* and *Bifidobacterium* were well tolerated and not associated with adverse events during the intervention period. The adverse events reported during treatment were gastrointestinal diseases, such as diarrhea, vomiting, etc., which also supported the findings of Allen et al. [47]. Although an increasing number of scholars [48] have discussed that the live microorganisms contained in breast milk may have important implications for the health of infants in the early stages of life, indicating that research of probiotics for infant foods should be given more attention, there is still a lack of clinical evidence on the safety of long-term use of probiotics and standard treatment prescriptions.

The capability of probiotics to prevent the development of eczema remains controversial. Unlike other studies, this meta-analysis only included trials of supplementing mixed strains of *Lactobacillus* and *Bifidobacterium*, and was aimed at infants under three years of age. We provided further evidence that *Lactobacillus* and *Bifidobacterium* mixed strains were indeed effective interventions to reduce the prevalence of eczema in infants. Considering that the effect of probiotics on allergic diseases is still uncertain, further research on their mode of action is needed.

The sensitivity analysis showed that Allen et al. [31] and Soh et al. [39] were considered as the source of the heterogeneity. The studies mentioned above yielded negative results. Soh et al. [39] did not observe a protective effect of probiotics on eczema which may have been due to the absence of prenatal probiotic supplementation in its protocols. However, in Allen et al.’s [31] study, four probiotic strains were used, which were redundant to other studies in meta-analysis. These are the reasons for the possible heterogeneity. Another source of heterogeneity may also be associated with trials that include probiotics or formula supplements that have been previously utilized by other reviews and meta-analyses [41].

This study has some limitations. First, we still cannot confirm that evidence can be covered due to the undetermined unpublished data. The selection and extraction of data may also cause some deviations. Second, different diagnostic criteria may affect the extrapolation of the results to a certain extent. Third, the included literature comes from randomized controlled trials performed in different countries. Children of different races and different environments will have various sensitivities and responses to probiotics at the same dose and concentration, which may affect the study results. Finally, although our research focused on infants under three years of age, the longest follow-up period in studies that met the inclusion criteria was two years after birth. We need more trials to prove the effectiveness of probiotics in preventing eczema in children aged 2–3 years old.

## 5. Conclusions

The meta-analysis showed that oral administration of mixed strains of *Lactobacillus* and *Bifidobacterium* to mothers during pregnancy has a positive effect on reducing infant eczema under three years of age. However, simply intervening with the infants after delivery showed no effect, which may be ascribed to the sample size, and this does not mean that it was useless. Further research is needed to explore the exact mechanism of the mixed strains of *Lactobacillus* and *Bifidobacterium* affecting infantile eczema.

## Figures and Tables

**Table 1:** Characteristics of the included trials.

Trial	Sample Size	Eczema Cases	Family History (Negative/Positive)	Adverse Events Cases	Intervention vs. Placebo	Specific Strains and Dosage (cfu/Day)	Intervention Time (Start–End)	Follow-Up Time
Allen 2014	T: 214 C: 222	T: 73 C: 72	Negative	T: 0 C: 0	*Lactobacillus* and *Bifidobacterium* vs. Maltodextrin	*Lactobacillus salivarius* (*L. salivarius*) (6.25 × 10^9^), *L. paracasei* (1.25 × 10^9^), *Bifidobacterium animalis subspecies lactis* (*B. animals subsp. lactis*) (1.25 × 10^9^), *Bifidobacterium bifidum* (*B. bifidum*) (1.25 × 10^9^)	Mother: from gestational week 36 until delivery Infants: 0–6 months of age	2 years
Dotterud 2010	T: 138 C: 140	T: 29 C: 48	Negative	T: 0 C: 0	*Lactobacillus* and *Bifidobacterium* vs. Skim fermented milk	*LGG* (5 × 10^10^), *B. animals subsp. lactis* (5 × 10^10^), *Lactobacillus acidophilus* (*L.acidophilus*) (5 × 10^9^)	Mother: 36 weeks of pregnancy–3 months postpartum	2 years
Huurre 2008	T: 72 C: 68	T: 7 C: 12	Positive	Not report.	*Lactobacillus* and *Bifidobacterium* vs. Microcrystalline cellulose and anhydrous glucose	*LGG* (1 × 10^10^), *B. animals subsp. lactis* (1 × 10^10^)	Mother: the first trimester of pregnancy–the end of exclusive breastfeeding	1 year
Kim 2010	T: 33 C: 35	T: 12 C: 22	Positive	T: 0 C: 0	*Lactobacillus* and *Bifidobacterium* vs. Maltodextrin	*B. bifidum* (1.6 × 10^9^), *B. animals subsp. lactis* (1.6 × 10^9^), *L. acidophilus* (1.6 × 10^9^)	Mother: 8 weeks before delivery–6 months after delivery	1 year
Rautava 2012 (1)	T: 73 C: 62	T: 21 C: 44	Positive	T: 35 C: 24	*Lactobacillus* and *Bifidobacterium* vs. Dietary supplement without probiotics	*LGG* (1 × 10^9^), *B. longum* (1 × 10^9^)	Mother: 2 months before delivery–2 months after delivery	2 years
Rautava 2012 (2)	T: 70 C: 62	T: 20 C: 44	Positive	T: 28 C: 24	*Lactobacillus* and *Bifidobacterium* vs. Dietary supplement without probiotics	*L. paracasei* (1 × 10^9^), *B. longum* (1 × 10^9^)	Mother: 2 months before delivery–2 months after delivery	2 years
Ro 2017	T: 68 C: 72	T: 16 C: 28	Negative	Not report.	*Lactobacillus* and *Bifidobacterium* vs. placebo	*LGG* (5 × 10^10^), *B. animals subsp. lactis* (5 × 10^10^), *L. acidophilus* (5 × 10^9^)	Mother: 36 weeks of pregnancy–3 months postpartum	2 years
Schmidt 2019	T: 130 C: 130	T: 5 C: 14	Negative	T: 12 C: 13	*Lactobacillus* and *Bifidobacterium* vs. Maltodextrin	*LGG* (1 × 10^9^), *B. animals subsp. lactis* (1 × 10^9^)	Infant: 6 months	half a year
Simpson 2016	T: 129 C: 130	T: 29 C: 45	Negative	Not report.	*Lactobacillus* and *Bifidobacterium* vs. Fermented skimmed milk	*LGG* (5 × 10^10^), *B. animals subsp. lactis* (5 × 10^10^), *L. acidophilus* (5 × 10^9^)	Mother: 36 weeks of pregnancy–3 months postpartum	2 years
Soh 2009	T: 124 C: 121	T: 27 C: 30	Positive	Not report.	*Lactobacillus* and *Bifidobacterium* vs. infant formula	*B. longum* (1 × 10^7^), *LGG* (2 × 10^7^)	Infants: 0–6 months of age	1 year

*LGG*: *Lactobacillus rhamnosus GG*, T: treatment group, C: control group.

## Data Availability

Data are contained within the paper.

## Supplementary Materials

The following are available online at https://www.mdpi.com/article/10.3390/nu13051461/s1, Figure S1: Forest plot of the effect of probiotics dosage on occurrence of Infantile Eczema, Figure S2: Forest plot of the effect of probiotics strains on occurrence of Infantile Eczema, Table S1: Search strategy.
