# Supplementary material for: Can Mixed Strains of Lactobacillus and Bifidobacterium Reduce Eczema in Infants under Three Years of Age? A Meta-Analysis
Source: Nutrients. 2021 Apr 25;13(5):1461. doi: 10.3390/nu13051461 (PMC8145948; doi:10.3390/nu13051461)
Supplement: Supplementary file 1 [file nutrients-13-01461-s001.zip › nutrients-1177461-supplementary.pdf]

Supplementary Materials

Table S1. Search strategy (example for 3 databases).

Pubmed	Search terms for query	Results
#1	Search ((Probiotics[MeSH Terms]) OR Bifidobacterium[MeSH Terms]) OR Lactobacillus[MeSH Terms]	41150
#2	Search ((Eczema[MeSH Terms]) OR Allergic diseases[MeSH Terms]) OR Atopic dermatitis[MeSH Terms]	30229
#3	Search ((((Probiotics[MeSH Terms]) OR Bifidobacterium[MeSH Terms]) OR Lactobacillus[MeSH Terms])) AND (((Eczema[MeSH Terms]) OR Allergic diseases[MeSH Terms]) OR Atopic dermatitis[MeSH Terms])	373
#4	Search (((Infant[MeSH Terms]) OR Children[MeSH Terms]) OR Pregnancy[MeSH Terms]) OR Pregnant woman[MeSH Terms]	3138055
#5	Search ((((((Probiotics[MeSH Terms]) OR Bifidobacterium[MeSH Terms]) OR Lactobacillus[MeSH Terms])) AND (((Eczema[MeSH Terms]) OR Allergic diseases[MeSH Terms]) OR Atopic dermatitis[MeSH Terms]))) AND ((((Infant[MeSH Terms]) OR Children[MeSH Terms]) OR Pregnancy[MeSH Terms]) OR Pregnant woman[MeSH Terms])	239

Web of Science	Search terms for query	Results
#1	TI = (Probiotics OR Bifidobacterium OR Lactobacillus)<br>Timespan = Before January 2020; Search language=Auto	35582
#2	TI = (Eczema OR Allergic diseases OR Atopic dermatitis)<br>Timespan = Before January 2020; Search language=Auto	36223
#3	#1 AND #2<br>Timespan = Before January 2020; Search language=Auto	312
#4	TI = (Infant OR Children OR Pregnancy OR Pregnant woman)<br>Timespan = Before January 2020; Search language=Auto	1380268
#5	#3 AND #4<br>Timespan = Before January 2020; Search language=Auto	84

The Cochrane library	Search terms for query	Results
#1	(Probiotics) OR (Bifidobacterium) OR (Lactobacillus)	9318
#2	(Eczema) OR (Allergic diseases) OR (Atopic dermatitis)	11338
#3	#1 AND #2	491
#4	(Infant) OR (Children) OR (Pregnancy) OR(Pregnant woman)	188350
#5	#3 AND #4	376
#6	#5 AND Randomized controlled trials	325

Study or Subgroup	Probiotics Events	Probiotics Total	Control Events	Control Total	Weight	Risk Ratio M-H, Random, 95% CI
**1.5.1 Daily dose of each strain≤1x10^9**						
Rautava 2012 (1)	21	73	44	62	11.6%	0.41 [0.27, 0.60]
Rautava 2012 (2)	20	70	44	62	11.5%	0.40 [0.27, 0.60]
Schmidt 2019	5	130	14	130	4.6%	0.36 [0.13, 0.96]
Soh 2009	27	124	30	121	10.6%	0.88 [0.56, 1.39]
**Subtotal (95% CI)**		**397**		**375**	**38.3%**	**0.49 [0.32, 0.75]**
Total events	73		132			
Heterogeneity: Tau² = 0.11; Chi² = 8.53, df = 3 (P = 0.04); I² = 65%						
Test for overall effect: Z = 3.30 (P = 0.0010)						
**1.5.2 Daily dose of each strain >1x10^9**						
Allen 2014	73	214	72	222	13.8%	1.05 [0.81, 1.37]
Dotterud 2010	29	138	48	140	11.6%	0.61 [0.41, 0.91]
Huurre 2008	7	72	12	68	5.5%	0.55 [0.23, 1.32]
Kim 2010	12	33	22	35	9.6%	0.58 [0.34, 0.97]
Ro 2017	16	68	28	72	9.6%	0.61 [0.36, 1.01]
Simpson 2016	29	129	45	130	11.6%	0.65 [0.44, 0.97]
**Subtotal (95% CI)**		**654**		**667**	**61.7%**	**0.70 [0.54, 0.90]**
Total events	166		227			
Heterogeneity: Tau² = 0.05; Chi² = 9.81, df = 5 (P = 0.08); I² = 49%						
Test for overall effect: Z = 2.75 (P = 0.006)						
**Total (95% CI)**		**1051**		**1042**	**100.0%**	**0.60 [0.47, 0.78]**
Total events	239		359			
Heterogeneity: Tau² = 0.10; Chi² = 27.07, df = 9 (P = 0.001); I² = 67%						
Test for overall effect: Z = 3.93 (P < 0.0001)						
Test for subgroup differences: Chi² = 1.96, df = 1 (P = 0.16), I² = 48.9%						

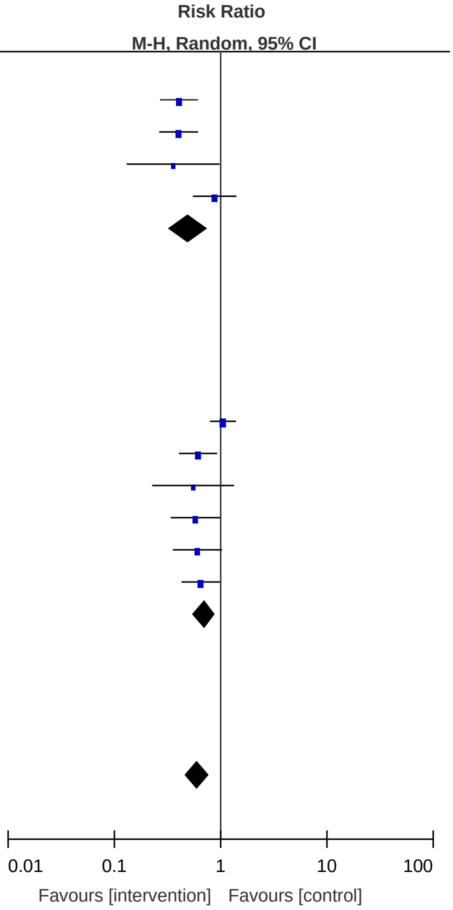

Figure S1. Forest plot of the effect of probiotics dosage on occurrence of Infantile Eczema.

Study or Subgroup	Probiotics Events	Probiotics Total	Control Events	Control Total	Weight	Risk Ratio M-H, Random, 95% CI
**1.6.1 LGG and B.longum**						
Rautava 2012 (1)	21	73	44	62	13.3%	0.41 [0.27, 0.60]
Soh 2009	27	124	30	121	12.0%	0.88 [0.56, 1.39]
**Subtotal (95% CI)**		**197**		**183**	**25.2%**	**0.59 [0.28, 1.27]**
Total events	48		74			
Heterogeneity: Tau^2 = 0.26; Chi^2 = 6.40, df = 1 (P = 0.01); I^2 = 84%						
Test for overall effect: Z = 1.35 (P = 0.18)						
**1.6.2 LGG、B.lactis and L. acidophilus**						
Dotterud 2010	29	138	48	140	13.2%	0.61 [0.41, 0.91]
Ro 2017	16	68	28	72	10.7%	0.61 [0.36, 1.01]
Simpson 2016	29	129	45	130	13.2%	0.65 [0.44, 0.97]
**Subtotal (95% CI)**		**335**		**342**	**37.2%**	**0.62 [0.49, 0.80]**
Total events	74		121			
Heterogeneity: Tau^2 = 0.00; Chi^2 = 0.06, df = 2 (P = 0.97); I^2 = 0%						
Test for overall effect: Z = 3.73 (P = 0.0002)						
**1.6.3 LGG and B.lactis**						
Huurre 2008	7	72	12	68	5.8%	0.55 [0.23, 1.32]
Schmidt 2019	5	130	14	130	4.8%	0.36 [0.13, 0.96]
**Subtotal (95% CI)**		**202**		**198**	**10.7%**	**0.46 [0.24, 0.88]**
Total events	12		26			
Heterogeneity: Tau^2 = 0.00; Chi^2 = 0.42, df = 1 (P = 0.52); I^2 = 0%						
Test for overall effect: Z = 2.35 (P = 0.02)						
**1.6.4 Other strains**						
Allen 2014	73	214	72	222	16.2%	1.05 [0.81, 1.37]
Kim 2010	12	33	22	35	10.7%	0.58 [0.34, 0.97]
Rautava 2012 (2)	20	70	44	0		Not estimable
**Subtotal (95% CI)**		**317**		**257**	**26.9%**	**0.81 [0.46, 1.46]**
Total events	105		138			
Heterogeneity: Tau^2 = 0.14; Chi^2 = 4.06, df = 1 (P = 0.04); I^2 = 75%						
Test for overall effect: Z = 0.69 (P = 0.49)						
**Total (95% CI)**		**1051**		**980**	**100.0%**	**0.64 [0.50, 0.82]**
Total events	239		359			
Heterogeneity: Tau^2 = 0.08; Chi^2 = 20.77, df = 8 (P = 0.008); I^2 = 61%						
Test for overall effect: Z = 3.48 (P = 0.0005)						
Test for subgroup differences: Chi^2 = 1.71, df = 3 (P = 0.64), I^2 = 0%						

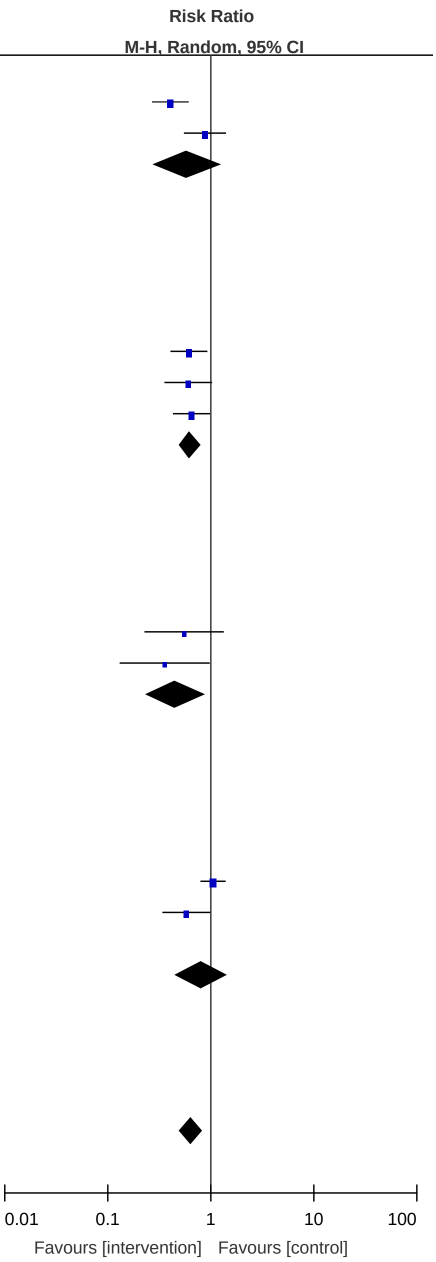

Figure S2. Forest plot of the effect of probiotics strains on occurrence of Infantile Eczema.
